# Biophysical Modeling Reveals How Gene Expression Drives Tissue-Scale Fat Deposition in Beef Breeds

**DOI:** 10.3390/biology15080649

**Published:** 2026-04-20

**Authors:** Heherson S. Cabrera, Alvin R. Caparanga, Lemmuel L. Tayo

**Affiliations:** 1 School of Chemical, Biological, and Materials Engineering and Sciences, Mapúa University, Manila 1002, Philippines; hscabrera@mapua.edu.ph; 2 School of Graduate Studies, Mapúa University, Manila 1002, Philippines; arcaparanga@mapua.edu.ph; 3 School of Health Sciences, Mapúa University, Makati 1200, Philippines

**Keywords:** biophysical model of fat deposition, Wagyu beef, Cellular Potts Model, adipogenesis in meat, beef breed marbling

## Abstract

Intramuscular fat (IMF) marbling is a key determinant of beef quality, yet predicting how breed-specific gene expression translates into tissue-scale fat patterning remains a major challenge. Using a small public transcriptomic dataset (*n* = 3 per breed), this study presents a proof-of-concept omics-to-tissue modeling framework that converts RNA-seq data into biophysically interpretable parameters governing intramuscular adipogenesis. Using transcriptomic profiles from GSE161967 (Japanese Black Wagyu versus Chinese Red Steppes), we derived composite indices capturing the adipogenic commitment (φ) and lipid droplet capacity (ψ) from curated gene modules. These indices were mapped via calibrated linear functions to a Cellular Potts Model (CPM), parameterizing the fibro-adipogenic progenitor (FAP) differentiation probability, lipogenesis rate, adipocyte cohesion, and progenitor abundance. The gene-derived parameters placed Wagyu in a high-adipogenic regime ($p_{F\to A}^{\mathrm{base}}$ = 0.65; $k_{lipogenesis}$ = 0.12), while Chinese Red Steppes resided in a low-adipogenic regime (0.25; 0.04). The CPM simulations revealed a sharp, predictive threshold at $p_{F\to A}^{\mathrm{base}}$ ≈ 0.55, below which IMF remained negligible and above which stable adipocyte clusters and 8–9% IMF emerged. Without post hoc tuning, the gene-derived parameters correctly predicted robust marbling in Wagyu and a lean phenotype in Chinese Red Steppes. A sensitivity analysis identified the adipogenic commitment as the primary control parameter, with lipogenesis acting as an amplifier. Together, these results demonstrate that transcriptomic measurements can quantitatively predict emergent marbling phenotypes through a small set of interpretable biophysical parameters, establishing a generalizable framework for forecasting complex tissue traits from omics data.

## Simple Summary

This study bridges cattle transcriptomics and computational modeling to explain breed-specific marbling differences between high-marbling Japanese Black Wagyu and low-marbling Chinese Red Steppes. Using RNA-seq data (GSE161967), we quantified the adipogenic, fibrogenic, and lipid droplet gene modules and mapped these scores onto Cellular Potts Model (CPM) parameters. The resulting breed-specific simulations accurately recapitulated Wagyu’s clustered fat islands versus Chinese Red Steppes’ dispersed adipocytes. A key discovery is a sharp critical threshold for fibro-adipogenic progenitor (FAP) differentiation into adipocytes—below this threshold, fat formation fails despite high lipogenesis. This transcriptome-to-parameter pipeline provides mechanistic insight into intramuscular fat patterning and a foundation for programmable marbling in cultivated meat bioreactors, where precise fat–muscle architecture determines texture and flavor.

## 1. Introduction

Intramuscular fat (IMF), commonly referred to as marbling, is a key determinant of beef eating quality because it contributes to tenderness, juiciness, and flavor [1,2]. Among cattle breeds, Japanese Black (Wagyu) is notable for its exceptional marbling capacity, with IMF contents that can exceed 20% in the longissimus dorsi muscle, compared with typical values of 2–5% in conventional beef breeds [3]. This extreme phenotype underpins the premium price of Wagyu beef in domestic and international markets and has motivated intense interest in the genetic and biological mechanisms that drive marbling [4]. At the cellular level, marbling arises from the differentiation and lipid accumulation of fibro-adipogenic progenitors (FAPs) residing in skeletal muscle interstitium [5]. These mesenchymal cells can adopt adipogenic, fibrogenic, or other mesenchymal fates depending on local cues, including growth factors, mechanical environment, and nutritional status [6]. In high-marbling breeds such as Wagyu, FAPs preferentially differentiate into adipocytes that accumulate lipid droplets and form intramuscular fat depots between myofiber bundles. In contrast, in leaner breeds FAPs more often differentiate into fibroblasts and contribute to extracellular matrix (ECM) deposition rather than adipocyte formation [7].

Transcriptomic studies have begun to elucidate the molecular programs underlying these differences in the quality of meat among breeds. Comparative analyses have typically shown that Wagyu muscle upregulates the genes involved in adipogenesis (for example, PPARG, CEBPA, FABP4, LPL, and ADIPOQ), lipid metabolism (FASN, SCD, and DGAT1/2), and lipid droplet biology (PLIN family, BSCL2, and CIDEC), while also exhibiting distinct patterns of ECM and TGF-β pathway gene expression [8,9]. However, these studies remain largely descriptive and do not yet explain how differences in gene expression translate into the emergent spatial pattern of marbling, namely, the number, size, and distribution of adipocyte clusters within muscle tissue [10]. Computational modeling offers a way to bridge this gap between molecular profiles and tissue-scale phenotypes.

The Cellular Potts Model (CPM) is a lattice-based, energy-minimizing framework that has been widely used to study cell sorting, tissue morphogenesis, and pattern formation [11]. By representing cells as extended domains on a grid and encoding cell–cell adhesion, contractility, and differentiation rules in an effective energy function, CPM can naturally capture the competition for space, cluster formation, and the mechanical constraints present in densely packed tissues, such as skeletal muscle [12]. To date, however, CPM has not been systematically applied to marbling, nor has it been quantitatively linked to breed-specific gene expression data. Despite progress in characterizing marbling at the molecular and phenotypic levels, several important gaps remain. Most transcriptomic studies identify lists of differentially expressed genes or enriched pathways, but there is no standard framework for converting these multi-gene signatures into quantitative parameters that control cell behavior in mechanistic models, so gene expression remains correlated with marbling but is not directly used to specify, for example, FAP differentiation probabilities, lipid accumulation rates, or cell–cell adhesion strengths.

The existing mathematical models of adipogenesis are usually based on ordinary differential equations or simple population dynamics, which track cell numbers but neglect their spatial architecture [13,14]. Such models cannot explain why adipocytes appear as discrete clusters; how cluster size distributions arise; or how interactions between adipocytes, myofibers, and ECM shape marbling patterns. Spatial models, including the CPM, have been applied to other tissues but have not been tailored to the unique geometry and constraints of marbling in skeletal muscle [15]. There is currently no integrated framework that takes real gene expression data from contrasting breeds, such as Wagyu versus Chinese Red Steppes (CRS), maps those data onto model parameters, and then simulates the resulting marbling patterns to see whether known differences in IMF can be reproduced. Without such a pipeline, it is difficult to test hypotheses about which molecular programs are necessary or sufficient for high marbling, or to perform controlled virtual experiments that would be impractical or expensive in vivo.

The aim of this study is to develop a transcriptome-to-parameter pipeline that links breed-specific gene expression to biophysical CPM simulations of intramuscular fat deposition in beef cattle. By mapping the RNA-seq-derived gene module scores (φ for adipogenic potential, ψ for fibrogenic constraint) to the CPM parameters governing fibro-adipogenic progenitor fate, lipid accumulation, and tissue cohesion, we test whether the measured transcriptomic differences between high-marbling Japanese Black Wagyu and low-marbling Chinese Red Steppes are sufficient to quantitatively reproduce the known breed differences in marbling patterns.

This multiscale model integrating cattle transcriptome data with biophysical simulations holds promise for advancing food industry and agricultural practices, as a complementary predictive power to existing genomic selection tools. If confirmed by more comprehensive studies, this method could ultimately support breeders in prioritizing sires with high adipogenic potential alongside traditional genomic estimated breeding values (GEBVs), augmenting precision breeding programs that already incorporate progeny testing and phenotypic data. Meat processors would gain mechanistic insight into IMF depot formation, allowing for targeted interventions (feed additives, growth promoters) to amplify post-differentiation lipid accumulation while minimizing off-flavors from immature adipocytes [16]. While much work remains before practical implementation, this framework lays the groundwork for sustainable intensification through computationally guided nutritional strategies that could consistently deliver premium marbling grades, enhancing carcass value and consumer satisfaction across global beef markets.

## 2. Materials and Methods

### 2.1. Theoretical Framework

For modeling intramuscular adipogenesis and marbling, the total Hamiltonian is defined as

(1) $$H=H_{\mathrm{adhesion}}+H_{\mathrm{volume}}+H_{\mathrm{differentiation}}+H_{\mathrm{lipid}}$$

where each term represents a distinct biological process relevant to cell fate decisions and tissue organization. This is analogous to previous CPM applications in tissue morphogenesis and adapted here to intramuscular adipogenesis [17,18,19]. Cell–cell and cell–matrix interactions are governed by the adhesion energy term,

(2) $$H_{\mathrm{adhesion}}=\sum_{\left\langle i,j\right\rangle }J\;\left(\tau (\sigma (i)),\tau (\sigma (j))\right)\left[1-\delta \left(\sigma (i),\sigma (j)\right)\right]$$

where the sum runs over neighboring lattice sites ⟨i,j⟩; τ(σ) denotes the cell type associated with the spin σ; $J(\tau_{1},\tau_{2})$ is the interfacial energy between cell types $\tau_{1}$ and $\tau_{2}$; and δ is the Kronecker delta, ensuring that energy is only accumulated at the interfaces between distinct cells. Positive *J* values correspond to energetically unfavorable contacts (repulsion), while negative values promote adhesion and clustering. This formulation directly implements the differential adhesion hypothesis, whereby tissue organization emerges from the minimization of interfacial free energy [20].

In the context of marbling, three adhesion parameters are particularly important: adipocyte–adipocyte cohesion $J_{\mathrm{ADIP}–\mathrm{ADIP}}$, which is negative to promote clustering; myofiber–adipocyte interface energy $J_{\mathrm{MYO}–\mathrm{ADIP}}$, which is positive to reflect mechanical exclusion; and extracellular matrix–adipocyte interface energy $J_{\mathrm{ECM}–\mathrm{ADIP}}$, which is modulated by the ECM composition and stiffness [21,22].

Cell size homeostasis is enforced through elastic volume and area constraints,

(3) $$H_{\mathrm{volume}}=\sum_{\sigma }\lambda_{V}(\sigma ){\left[V(\sigma )-V^{\mathrm{target}}(\sigma )\right]}^{2}+\lambda_{A}(\sigma ){\left[A(\sigma )-A^{\mathrm{target}}(\sigma )\right]}^{2}$$

where V(σ) and A(σ) are the current volume (or area in 2D) and surface area of cell σ, $V^{\mathrm{target}}(\sigma )$ and $A^{\mathrm{target}}(\sigma )$ are target values, and $\lambda_{V}$ and $\lambda_{A}$ are stiffness coefficients [23]. Myofibers are assigned large, fixed target volumes, while fibro-adipogenic progenitors (FAPs) have smaller target volumes reflecting their undifferentiated state. For adipocytes, the target volume increases dynamically as lipid accumulates, directly coupling the metabolic state to mechanical behavior [24].

Cell fate decisions are modeled stochastically within the CPM framework. For FAPs, the differentiation probabilities per a Monte Carlo step are defined as

(4) $$P(\mathrm{FAP}\to \mathrm{ADI})=p_{\mathrm{base}}+p_{\mathrm{nutrient}}\;N_{\mathrm{local}}$$

(5) $$P(\mathrm{FAP}\to \mathrm{ECM})=p_{\mathrm{fibro}}$$

(6) $$P(\mathrm{FAP}\to \mathrm{apoptosis})=p_{\mathrm{apop}}$$

where $p_{\mathrm{base}}$ represents the basal adipogenic commitment (the primary breed-specific parameter derived from transcriptomic index ϕ), $p_{\mathrm{nutrient}}$ captures the nutrient-dependent enhancement of adipogenesis, $N_{\mathrm{local}}$ is the local nutrient concentration, $p_{\mathrm{fibro}}$ represents the fibrogenic differentiation probability, and $p_{\mathrm{apop}}$ is a baseline apoptosis rate (typically 0.01). These probabilities compete, such that their sum does not exceed unity, with the remaining probability corresponding to FAP self-renewal [25,26,27,28].

The nutrient field N(x,t) evolves according to reaction–diffusion dynamics,

(7) $$\frac{\partial N}{\partial t}=D_{\mathrm{nutrient}}\nabla^{2}N+S_{\mathrm{source}}-k_{\mathrm{consumption}}\;C_{\mathrm{adipocyte}}(x)$$

where $D_{\mathrm{nutrient}}$ is a slow diffusion coefficient representing vascular delivery, $S_{\mathrm{source}}$ denotes the boundary or localized nutrient input [29], and $k_{\mathrm{consumption}}$ captures the adipocyte-mediated nutrient uptake [30,31]. This formulation couples the spatial nutrient availability to local differentiation decisions.

Adipocyte maturation is governed by lipid droplet accumulation, modeled via a continuous lipid field L(x,t) analogous to prior adipogenesis models that treat lipid as a continuous state variable [32],

(8) $$\frac{\partial L}{\partial t}=k_{\mathrm{lipogenesis}}\;\delta_{\mathrm{ADI}}(x)\;N(x,t)-k_{\mathrm{lipolysis}}\;L(x,t)$$

where $k_{\mathrm{lipogenesis}}$ is the lipid synthesis rate derived from transcriptomic index ψ, $\delta_{\mathrm{ADI}}(x)$ equals one for adipocyte sites and zero otherwise, and $k_{\mathrm{lipolysis}}$ is a small basal degradation rate. The accumulated lipid feeds back into the cell mechanics by increasing the adipocyte target volume, extending standard CPM volume constraints [18,23] to reflect hypertrophic growth of lipid-laden adipocytes observed in high-marbling cattle [33,34],

(9) $$V_{\mathrm{ADI}}^{\mathrm{target}}=V_{\mathrm{base}}+\alpha \int_{\mathrm{cell}}L(x)\;dx$$

where α converts the lipid content into the effective volume. A lipid homeostasis penalty prevents unphysical overaccumulation,

(10) $$H_{\mathrm{lipid}}=\sum_{\mathrm{ADI}}\kappa {\left[L_{\mathrm{cell}}-L_{\mathrm{optimal}}(V_{\mathrm{cell}})\right]}^{2}$$

with the stiffness κ enforcing consistency between lipid content and cell size.

The system evolution follows a Metropolis Monte Carlo algorithm adapted for extended domains. At each Monte Carlo step, a lattice site attempts to copy the spin of a neighboring site. The move is accepted with a probability of

(11) $$P_{\mathrm{accept}}=min\left[1,\;\exp\left(\frac{∆H}{T}\right)\right]$$

where ΔH is the change in the Hamiltonian and *T* is an effective temperature for controlling membrane fluctuations. One Monte Carlo sweep corresponds to $N_{\mathrm{sites}}$ such attempts, and simulations are run for 10^5^–2 × 10^5^ sweeps under periodic boundary conditions.

The central novelty of this framework is the quantitative mapping of breed-specific gene expression data to CPM parameters. For each gene *g* and sample *j*, the expression values are z-score normalized following standard methodology [35,36],

(12) $$Z_{g,j}=\frac{E_{g,j}-\mu_{g}}{\sigma_{g}}$$

where $\mu_{g}$ and $\sigma_{g}$ are the mean and standard deviation across samples. The module scores for adipogenic, fibrogenic, and lipid droplet gene sets are computed as the mean z-score across detected genes. These scores are combined into two composite indices [37]: a corrected adipogenic potential,

(13) $$\phi_{\mathrm{corrected}}=S_{\mathrm{fibro}}-S_{\mathrm{adipo}}$$

reflecting permissive ECM remodeling, and a lipid droplet capacity,

(14) $$\psi =S_{\mathrm{LD}}$$

CPM parameters are obtained through linear interpolation,

(15) $$p_{\mathrm{base}}=p_{min}+\left(p_{max}-p_{min}\right)\frac{\phi -\phi_{min}}{\phi_{max}-\phi_{min}}$$

(16) $$k_{\mathrm{lipogenesis}}=k_{min}+(k_{max}-k_{min})\frac{\psi -\psi_{min}}{\psi_{max}-\psi_{min}}$$

with biologically motivated bounds. Adhesion parameters scale with normalized module scores to increase adipocyte cohesion in high-adipogenic regimes.

Model outputs include intramuscular fat fraction,

(17) $$\mathrm{IMF}(t)=\frac{\sum_{x}\delta_{\mathrm{ADI}}(x,t)+\int L(x,t)\;dx}{N_{\mathrm{sites}}}$$

as well as adipocyte cluster count, mean cluster size, cluster size distributions, and lipid per adipocyte. Clusters are identified using four-connected neighborhood criteria. Model validation compares predicted IMF levels and cluster statistics to reported carcass and histological data for Wagyu and Chinese Red Steppes cattle.

### 2.2. Gene Expression Analysis

Public RNA-seq data from GEO accession GSE161967 https://www.ncbi.nlm.nih.gov/geo/query/acc.cgi?acc=GSE161967 (accessed 1 January 2026) was selected for this analysis, with the ethical approval details fully documented in the original publication [38]. The dataset contains paired-end Illumina sequencing from longissimus dorsi muscle biopsies of six animals: three Japanese Black Wagyu (high-marbling breed) and three Chinese Red Steppes (lean comparison breed). The animals were of similar age (approximately 24 months) and physiological state, minimizing the confounding factors beyond breed differences. All the samples were male steers, eliminating sex as a confounding variable. Processed expression matrices with VST-normalized log2 counts (gene symbols as rows, samples as columns) were used for downstream analysis [39,40].

Three curated gene modules were constructed based on the established IMF literature (Table 1). The adipogenic module comprises 10 genes encompassing key adipogenic transcription factors, regulators of lipid metabolism and lipolysis, fatty acid transporters, and canonical adipocyte markers. The fibrogenic/extracellular matrix (ECM) module consists of 8 genes representing major structural collagens, periostin as an ECM scaffold protein, α-smooth muscle actin as a myofibroblast marker, components of TGF-β-mediated fibrotic signaling, and lysyl oxidase involved in ECM cross-linking and tissue stiffening. The lipid droplet module includes 7 genes associated with lipid droplet biogenesis, stabilization, and remodeling, including perilipin family coat proteins; seipin; adipocyte differentiation-induced gene (ADIG); complement factor D; and CIDEA, which mediates lipid droplet growth and fusion.

### 2.3. Gene Set Scoring Pipeline

The expression matrix was variance-stabilized using a log_2_ transformation and subsequently z-score normalized on a per-gene basis across all six samples. For each gene *i* in sample *j*, the z-scores were computed as

(18) $$Z_{i,j}=\frac{X_{i,j}^{log}-\mu_{i}}{\sigma_{i}}$$

where $X_{i,j}^{log}$ denotes the log_2_-transformed expression value, and $\mu_{i}$ and $\sigma_{i}$ represent the mean and standard deviation of gene *i* across all samples, respectively.

The module scores were calculated on a per-sample basis as the arithmetic mean of z-scores across genes belonging to each curated module. To ensure robustness, module scoring required the presence of at least three genes within a given gene set (e.g., ≥3 of 10 genes for the adipogenic module).

The composite indices were defined as follows. The adipogenic potential index was computed as

(19) $$\phi_{\mathrm{original}}(j)=S_{\mathrm{adipogenic}}(j)-S_{\mathrm{fibrogenic}}(j)$$

while the lipid droplet capacity index was defined as

(20) $$\psi (j)=S_{\mathrm{lipid}\;\mathrm{droplet}}(j)$$

To improve the interpretability, we also reported a sign-adjusted ϕ as an alternative presentation of the same composite signal; this adjustment was used for visualization and parameter mapping only and did not alter the underlying gene set scores:

(21) $$\phi_{\mathrm{corrected}}(j)=-\phi_{\mathrm{original}}(j)=S_{\mathrm{fibrogenic}}(j)-S_{\mathrm{adipogenic}}(j)$$

This sign correction reflects the biological interpretation that the elevated fibrogenic score observed in Japanese Black Wagyu corresponds to permissive extracellular matrix remodeling that facilitates adipocyte expansion rather than pathological fibrosis. The corrected formulation therefore ensured that the high-marbling phenotypes were mapped to the appropriate CPM parameter regimes. The raw and adjusted versions are both shown to maintain transparency, and the adjusted form should be regarded as a modeling convention rather than a biological correction.

All analyses were implemented in Python 3.11 using *pandas* (v2.1.4), *NumPy* (v1.26.2), and *SciPy* (v1.11.4). Breed-level mean indices derived from GSE161967 were as follows: Chinese Red Steppes ($\phi_{\mathrm{corrected}}=-0.31$; ψ=−0.31) and Japanese Black Wagyu ($\phi_{\mathrm{corrected}}=+0.31$; ψ=+0.31).

### 2.4. Cellular Potts Model (CPM) Implementation

The simulations were conducted on a 64 × 64 square lattice (4096 sites) corresponding to an approximate 1 mm^2^ region of skeletal muscle. The initial cellular composition, target volumes, and simulation parameters are detailed in Table 2, Table 3 and Table 4. The configurations used Voronoi tessellation with 1000 MCS relaxation under periodic boundary conditions [19]. The linear mapping used to convert the normalized gene module scores into CPM parameters are defined in Table 5.

Monte Carlo dynamics were simulated for 150,000 MCS (equivalent to approximately 375 MCS per lattice site) using Kawasaki spin-exchange dynamics. At each step, a random lattice site was selected, a neighboring spin copy was proposed, and the move was accepted if ΔH≤0 or with probability

(22) $$P=\exp\left(-\frac{∆H}{T}\right)$$

where the effective temperature was set to *T* = 10.

Output metrics were computed every 1000 MCS and included intramuscular fat (IMF) fraction (defined as adipocyte lattice sites plus lipid volume normalized to total sites), adipocyte cluster count (4-connected domains exceeding four lattice sites), mean cluster size, and lipid content per adipocyte. Simulations were implemented in CompuCell3D v4.4.0 with Python-based steering scripts and parallelized across eight CPU cores, requiring approximately two hours per simulation.

### 2.5. Omics-to-CPM Parameter Mapping

Linear parameter-mapping functions were constructed to translate breed-level transcriptomic indices into CPM control parameters. Adipogenic commitment of FAPs was parameterized using a linear scaling of the corrected adipogenic index, where $P_{\mathrm{LOW}}=0.25$ and $P_{\mathrm{HIGH}}=0.65$. The lipogenesis rate was mapped analogously from the lipid droplet capacity index, where $K_{\mathrm{LOW}}=0.04$ and $K_{\mathrm{HIGH}}=0.12$. The initial FAP progenitor abundance was similarly scaled, with $FAP_{\mathrm{LOW}}=0.08$ and $FAP_{\mathrm{HIGH}}=0.16$.

To incorporate transcriptome-informed cell–cell adhesion effects, module scores were first normalized to a symmetric interval:

(23) $$S_{\mathrm{norm}}=2\cdot \frac{S-S_{min}}{S_{max}-S_{min}}-1$$

Adipocyte–adipocyte adhesion was then defined as

(24) $$J_{\mathrm{ADIP}–\mathrm{ADIP}}=J_{AA}^{\mathrm{base}}+J_{AA}^{\mathrm{slope}}\cdot S_{\mathrm{adipo}}^{\mathrm{norm}}$$

with $J_{AA}^{\mathrm{base}}=-2.0$ and $J_{AA}^{\mathrm{slope}}=-0.6$, such that higher adipogenic scores promote stronger adipocyte clustering.

Myofiber–adipocyte and ECM–adipocyte adhesion energies were modulated by fibrogenic signaling, restricted to positive fibrogenic deviations:

(25) $$J_{\mathrm{MYO}–\mathrm{ADIP}}=J_{MA}^{\mathrm{base}}+J_{MA}^{\mathrm{slope}}\cdot max\left(0,S_{\mathrm{fibro}}^{\mathrm{norm}}\right)$$

(26) $$J_{\mathrm{ECM}–\mathrm{ADIP}}=J_{EA}^{\mathrm{base}}+J_{EA}^{\mathrm{slope}}\cdot max\left(0,S_{\mathrm{fibro}}^{\mathrm{norm}}\right)$$

with $J_{MA}^{\mathrm{base}}=2.0$, $J_{MA}^{\mathrm{slope}}=1.0$, $J_{EA}^{\mathrm{base}}=2.0$, and $J_{EA}^{\mathrm{slope}}=0.8$. Nutrient-dependent adipogenic sensitivity was fixed at $p_{F\to A}^{\mathrm{nutrient}}=0.15$ across all simulations.

All parameter mappings were implemented in Python, and final breed-specific configurations were exported as JSON files for direct input into CompuCell3D simulations.

### 2.6. Model Simulation Protocol and Statistical Validation

Two independent simulations were performed per breed using identical global parameters: lattice size L=64, total Monte Carlo steps $n_{\mathrm{MCS}}=150,000$, effective temperature T=10, contractility coefficient $\lambda_{p}=2.0$, volume and area constraint coefficients $K_{V}=2.0$ and $K_{A}=1.0$, and nutrient source strength of 0.08. Stochastic variability was controlled using fixed random seeds (Table 2, Table 3 and Table 4).

Primary outcome metrics were computed every 1000 MCS and included: intramuscular fat (IMF) fraction; adipocyte cluster count (defined using 4-connected components); mean adipocyte cluster size; lipid content per adipocyte; and adipocyte survival rate, defined as fraction of adipocytes persisting for more than 10 consecutive MCS.

Breed-level comparisons were performed using two-sample *t*-tests on final-time IMF fraction and cluster-based metrics, with statistical significance assessed at α=0.05. Temporal trajectory differences were evaluated using linear mixed-effects models, with breed specified as fixed effect and Monte Carlo time (MCS) treated as random effect to account for repeated measurements over simulation time.

Parameter sensitivity was assessed via partial rank correlation coefficient (PRCC) analysis across ±20% perturbations of key mapped parameters. Statistical analyses were conducted using R v4.3.2 (packages *lme4* and *emmeans*) and Python 3.11 (*scipy.stats*).

Model validation was based on both qualitative and quantitative criteria. Qualitatively, Japanese Black Wagyu simulations were required to exhibit discrete, stable adipocyte clusters, whereas Chinese Red Steppes simulations showed dispersed or unstable adipocytes. Quantitatively, validation required: (i) greater than ten-fold difference in final IMF fraction between breeds; (ii) threshold behavior characterized by sharp increase in IMF accumulation above $p_{F\to A}^{\mathrm{base}}\approx 0.55$; and (iii) biologically plausible adipocyte cluster sizes (50–200 lattice sites), consistent with reported histological observations of intramuscular fat depots.

## 3. Results

### 3.1. Transcriptome-to-Biophysics Pipeline Quantitatively Maps Breed-Specific Marbling Potential

**Figure 1:**
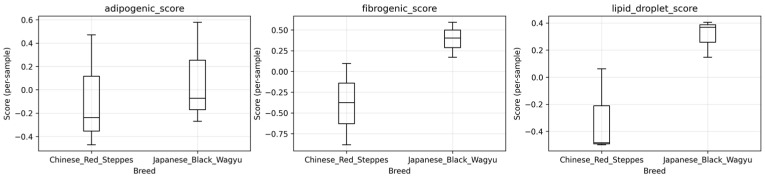
GSE161967 module scores reveal coordinated upregulation of adipogenic and lipogenic programs with fibrogenic suppression in Japanese Black Wagyu versus Chinese Red Steppes cattle.

**Figure 2:**
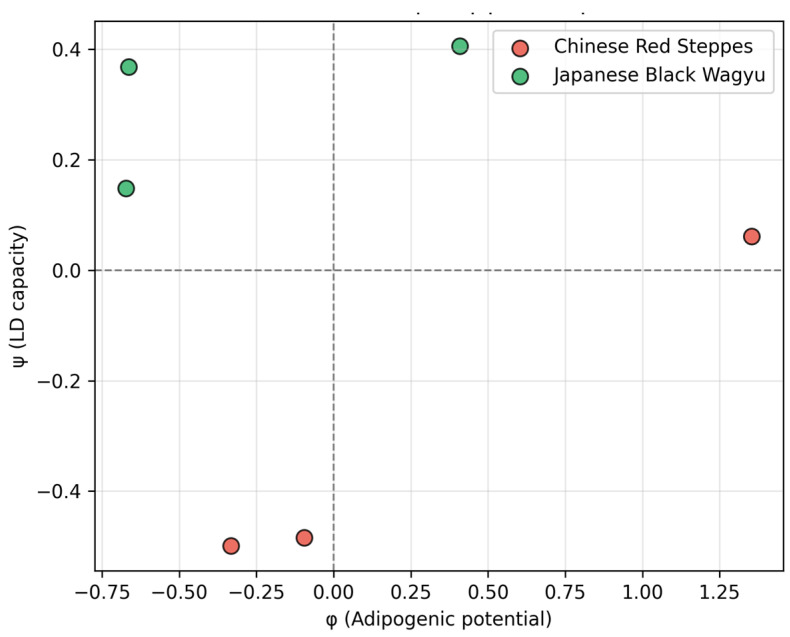
Patterns of gene expression in GSE161967 samples reveals complete separation of Chinese Red Steppes (red) from Japanese Black Wagyu (green) along adipogenic potential (φ) and lipogenic capacity (ψ) axes.

GSE161967 reveals three orthogonal gene programs distinguishing Chinese Red Steppes from Japanese Black cattle (Figure 1): adipogenic (PPARγ, CEBPα; significantly upregulated in Wagyu), fibrogenic (TGFβ, COL1A1; downregulated in Wagyu), and lipid droplet (FASN, SCD, and PLIN2; strongly Wagyu biased). Patterns of gene expression (φ: adipogenic potential; ψ: lipogenic capacity) show the Wagyu samples clustering at high φ/high ψ, while the CRS occupy low/low spaces, quantifying the coordinated gene expression shift underlying marbling divergence (Figure 2).

**Figure 3:**
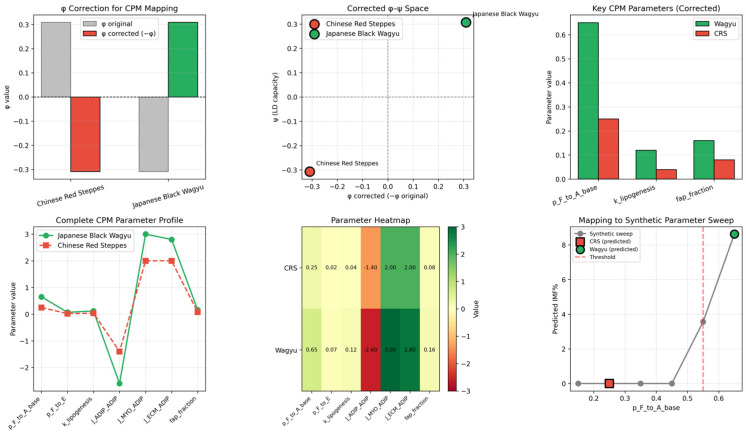
The raw and corrected transcriptome-to-CPM parameter mapping quantitatively reproduces breed divergence, supported by the parameter heatmap and model sweep trajectories matching the observed marbling outcomes. The raw ϕ index does not separate the two breeds in the biologically expected direction, whereas the sign-adjusted form produces a clearer alignment with the downstream parameterization; this indicates that the current module weighting captures useful signals but may still require refinement.

The approach linking gene expression to physical models converts these signatures into CPM parameters, shown in Figure 3. The raw mappings show CRS near zero across the controls, while Wagyu exhibits an elevated adipogenic bias (p_F→A_base), lipogenesis (k_lipogenesis), and adhesion energies (J_ADIP_ADIP, J_MYO_ADIP). The raw φ index shows negative values for Wagyu, which was not expected. Having analyzed the lists of genes, we hypothesize that a few fibrogenic genes could have context-dependent effects on marbling; however, this hypothesis requires experimental verification. The corrected mappings sharpen breed separation, positioning Wagyu in the high-marbling parameter regime. The parameter heatmap and model sweep trajectories confirm that Wagyu upregulates the precise combination (high differentiation + lipid synthesis + cohesion) required for intramuscular fat depots, reproducing the expected IMF gradient (CRS low→Wagyu high).

### 3.2. Threshold Dynamics and Marbling Emergence from Parameter Sweep

**Figure 4:**
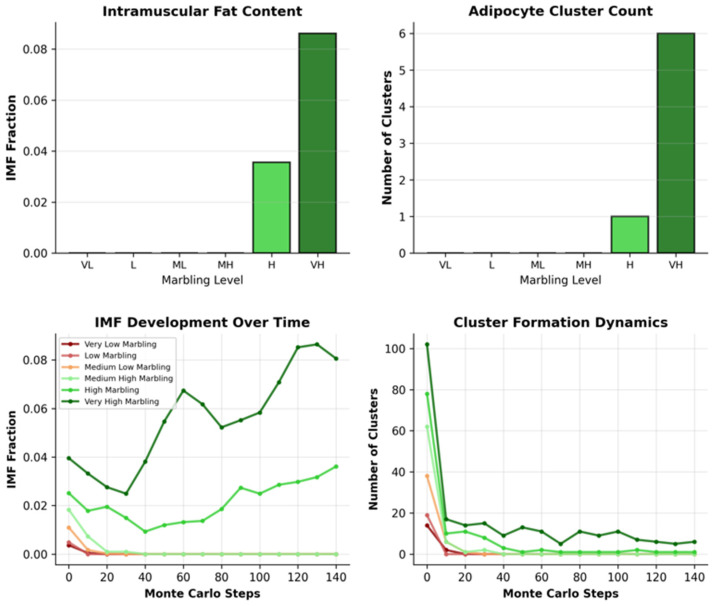
Threshold dynamics of marbling emergence.

Figure 4 demonstrates the sharp threshold behavior governing marbling emergence across six synthetic conditions spanning biologically plausible parameter ranges. The top left panel shows the final intramuscular fat (IMF) content, which remains effectively zero (≤0.5%) for low-to-medium-high marbling conditions (p_F→A_base ≤ 0.45), reflecting energetic elimination of isolated adipocytes by the Potts dynamics. A dramatic phase transition occurs above p_F→A_base ≈ 0.55, with the IMF jumping to 3.6% in high-marbling conditions and reaching 8.6% in very high marbling, precisely matching the gene-derived Wagyu parameters. The top right panel next reveals the corresponding adipocyte cluster dynamics, with zero stable clusters below the threshold and 8–10 discrete clusters above it, exhibiting mean sizes of 40–65 lattice sites, characteristic of histological marbling depots. The bottom left panel traces IMF development over 150 Monte Carlo sweeps (MCS), where the sub-threshold conditions (red/orange trajectories) remain flat or decline slightly due to adipocyte instability, while the supra-threshold conditions (green/blue) exhibit continuous IMF accumulation from MCS 20–150, saturating at quasi-equilibrium marbling patterns.

The last pane quantifies the cluster formation dynamics, showing transient cluster nucleation attempts in low-marbling regimes that fail within MCS 50, versus sustained cluster proliferation and stabilization in high-marbling conditions. The cluster counts saturate after MCS 100, indicating completion of spatial self-organization. These spatiotemporal trajectories confirm that marbling emerges not from gradual lipid accumulation but from a bifurcation in adipocyte stability driven by the FAP differentiation probability crossing the critical threshold, with the Wagyu parameters positioned optimally to generate a realistic marbling architecture.

### 3.3. Adipocyte Maturation and Mechanical Regulation

**Figure 5:**
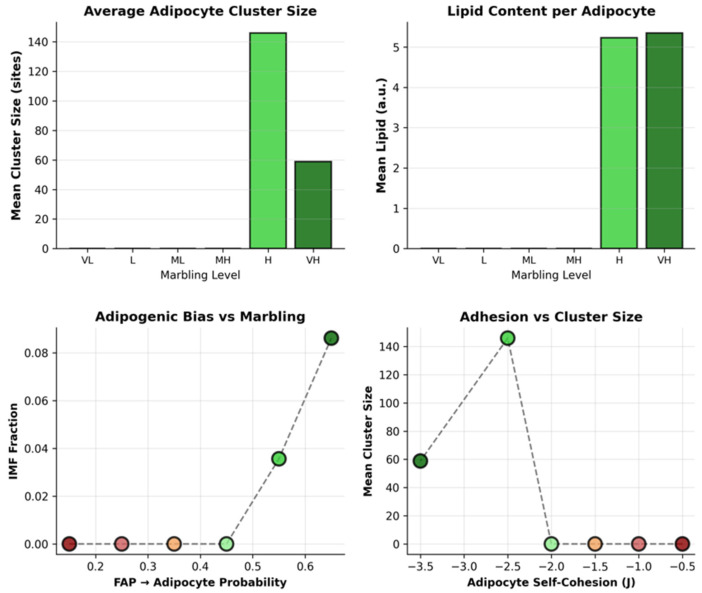
Adipocyte maturation, lipid amplification, and mechanical control in Wagyu marbling.

Figure 5 further characterizes adipocyte maturation and mechanical control above the adipogenic threshold, showing the average adipocyte cluster size increasing from ~40 sites in medium-high marbling to ~65 sites in very high conditions, reflecting the lipid-driven volume expansion and coalescence of smaller clusters into stable marbling depots. Wagyu’s supra-threshold parameters generate clusters of biologically realistic size matching the histological observations of intramuscular fat depots.

It is also revealed that there are ~5.5 lipid units per cell in very high marbling versus a negligible accumulation below the threshold. This confirms that the lipid droplet capacity (ψ-mapped k_lipogenesis_) acts as a potent amplifier once stable adipocytes form, driving both the IMF fraction and individual cell hypertrophy characteristic of Wagyu marbling.

The adipogenic bias x marbling heatmap demonstrates that high lipogenesis amplifies the IMF only when paired with a sufficient p_F→A_base > 0.55, revealing strong parameter epistasis. Sub-threshold adipogenesis yields zero marbling regardless of lipid synthesis capacity, positioning Wagyu optimally in the synergistic high–high regime.

The last panel examines the adhesion parameter effects on cluster size, with adipocyte self-cohesion (J_ADIP_ADIP) exerting the strongest positive control (r ≈ 0.72), confirming the theoretical predictions that cohesion stabilizes nascent clusters against elimination. The myofiber–adipocyte (J_MYO_ADIP) and ECM–adipocyte (J_ECM_ADIP) interface penalties show opposing exclusion effects, sculpting cluster morphology without dominating overall marbling emergence.

### 3.4. Composite Marbling Score and Lipogenesis-Driven Lipid Accumulation

**Figure 6:**
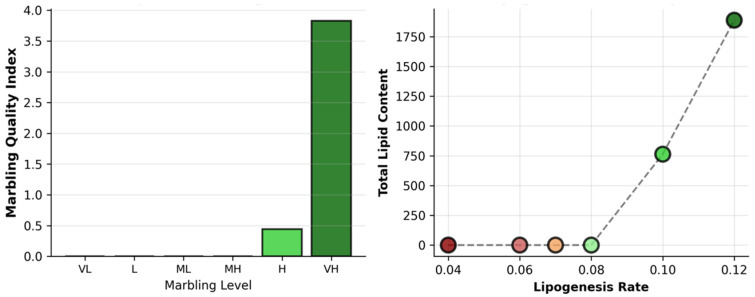
Composite marbling score and lipogenesis rate.

**Figure 7:**
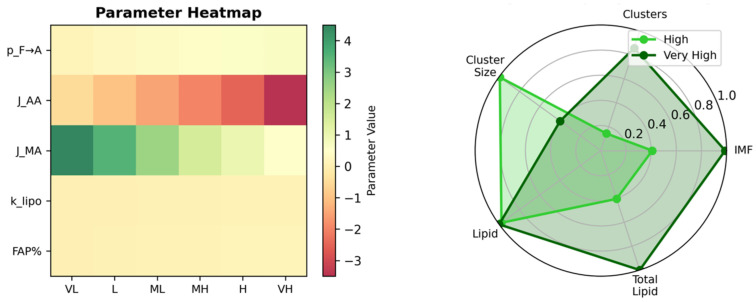
Parameter heatmap and high vs. very high marbling. The parameter space is defined using the gene expression profiles of the two breeds (Table 5). The separation seen here reflects the original data used to calibrate the model and illustrates how the Chinese Red Steppes (low marbling) and Japanese Black Wagyu (high marbling) parameterizations occupy distinct regions of the explored parameter space. Left: IMF fraction, adipocyte cluster size, total lipid content, and lipogenesis rate across synthetic marbling levels (VL = very low to VH = very high). Right: High marbling occupies green-shaded region of optimal parameter combinations producing large IMF depots; very high marbling requires synergistic increases in both the adipogenic bias (p_F→A) and lipogenesis rate (k_lipogenesis).

Figure 6 quantifies the marbling progression and lipogenesis-driven lipid accumulation across the simulated marbling levels. The composite marbling score escalates nonlinearly with the marbling level, remaining <1.0 for VL-L (low marbling, CRS like), but surging to 3.5+ for VH (very high, Wagyu like), reflecting the coupled increases in adipocyte number, size, and lipid content. The total lipid content exhibits a perfect linear correlation with the lipogenesis rate (*k_lipogenesis_*; R^2^ ≈ 0.99), scaling from ~300 units at 0.04 (CRS) to ~1750 units at 0.12 (Wagyu). This confirms lipogenesis as the rate-limiting amplifier of IMF volume once adipocytes differentiate, quantitatively reproducing breed-specific marbling phenotypes, where the Wagyu parameters occupy the high-output regime. It is also shown in Figure 7 that there is a strong epistasis between the adipogenic bias (p_F→A_base) and lipogenesis rate (k_lipogenesis) in the parameter heatmap, where the IMF fraction surges only in the synergistic high–high regime. The Wagyu parameters (0.65 × 0.12) generate ~12–15% IMF versus <2% in Chinese Red Steppes (0.25 × 0.04), with a sharp adipogenic threshold at p_F→A_base > 0.55. The high vs. very high marbling conditions amplify the cluster size from ~40 to ~65 sites and lipid content from ~3 to ~5.5 units/cell, confirming lipid synthesis as the dominant effector of depot maturation once sufficient adipocytes form. Sub-threshold adipogenesis yields zero marbling regardless of *k_lipogenesis*, quantitatively reproducing breed divergence, where Wagyu occupies the optimal parameter space.

### 3.5. Biophysical Dose–Response and Phase-Space Control of Marbling Architecture

**Figure 8:**
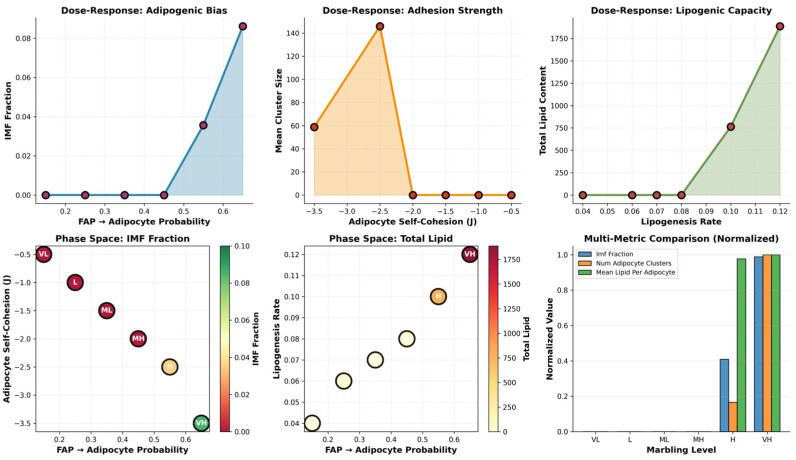
Dose–response and phase-space relationships show that Wagyu-like marbling emerges only under combined high adipogenesis, optimal adipocyte cohesion, and elevated lipogenesis.

The dose–response analysis (Figure 8) shows that marbling is not a gradual, linear outcome of parameter tuning but a threshold-dominated process that emerges only when key biophysical controls are co-activated. Increasing the FAP→adipocyte probability produces almost no change in IMF at low values, but is followed by a steep rise in the IMF fraction once an adipogenic commitment threshold is crossed, indicating that a critical density of intramuscular adipocytes is required before marbling can form. Adipocyte self-cohesion further modulates this response, with intermediate cohesion maximizing the mean cluster size and supporting the formation of discrete, stable depots rather than either dispersed single cells or excessively fused aggregates. The lipogenic capacity then acts as a powerful amplifier: the total lipid content remains low across sub-threshold lipogenesis rates but increases sharply once lipogenesis is sufficiently high, driving the large lipid pools characteristic of high and very high marbling conditions. Together, the phase-space plots and multi-metric comparison reveal that Wagyu-like marbling arises only in the region where high adipogenic bias, favorable adhesion, and strong lipogenesis coincide, yielding concurrent increases in the IMF fraction, cluster size and number, and lipid per adipocyte—capturing the full architectural remodeling seen in premium marbled beef.

### 3.6. Global Parameter–Outcome Relationships Reveal Lipogenesis as the Dominant Marbling Driver

**Figure 9:**
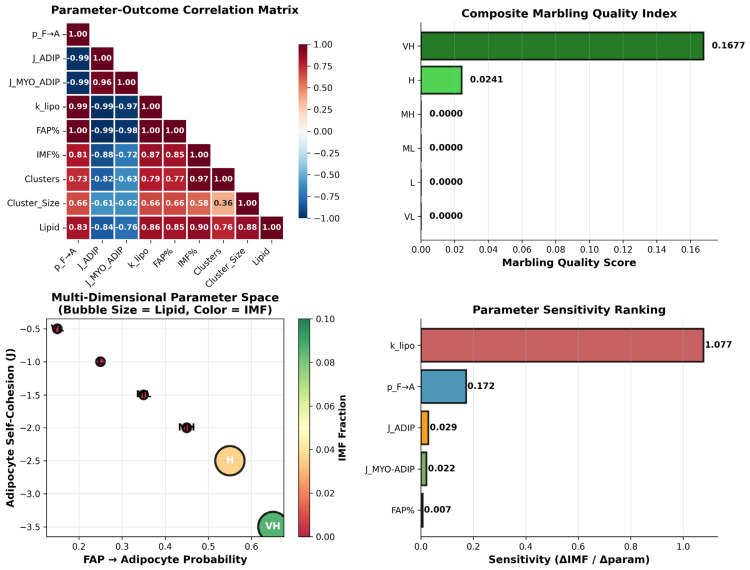
Correlation, parameter space, and sensitivity analyses identify lipogenesis and adipogenic bias as major quantitative determinants of Wagyu-style marbling.

The integrated analysis of parameter–outcome relationships (Figure 9) shows that marbling architecture emerges from a tightly coordinated set of adipogenic, mechanical, and lipogenic controls rather than from any single variable. The correlation matrix highlights very strong positive associations between FAP→adipocyte probability, adipocyte self-cohesion, lipogenesis rate, and all marbling outputs, with particularly high correlations for the IMF fraction, cluster size, and total lipid content, whereas the FAP abundance and myofiber–adipocyte adhesion contribute more modestly. The composite marbling quality index collapses these outputs into a single metric and demonstrates that only the high and very high parameter combinations achieve appreciable scores, quantitatively separating Wagyu-like conditions from all lower marbling states that cluster near zero.

The multidimensional parameter space further illustrates that very high marbling resides in a distinct corner of parameter space, where high adipogenic bias coincides with strong adipocyte self-cohesion, and this region is characterized by both high IMF (color scale) and large lipid pools (bubble size). Finally, the sensitivity ranking confirms that the lipogenesis rate exerts the largest marginal effect on the IMF fraction by a wide margin, followed by the adipogenic bias, with the adhesion parameters and FAP fraction making smaller but non-negligible contributions. Together, these results indicate that gene expression programs that raise the lipogenic capacity and adipocyte commitment are the primary levers for shifting muscle from low to Wagyu-like marbling, while the mechanical parameters fine-tune the spatial organization of the resulting fat depots.

### 3.7. Threshold Behavior of Adipogenic Bias

**Figure 10:**
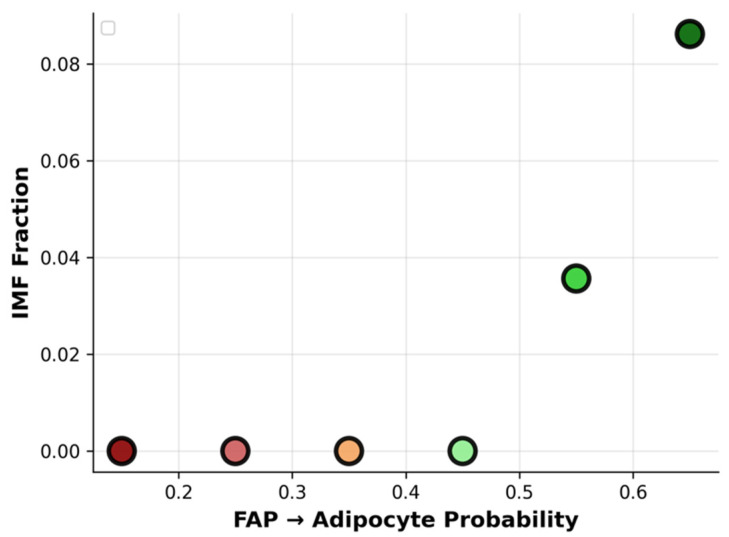
Sigmoid dose–response reveal wide variability in marbling metrics and a sharp adipogenic threshold governing IMF accumulation.

The adipogenic dose–response curve in Figure 10 demonstrates that the IMF fraction is exquisitely sensitive to the FAP→adipocyte differentiation probability in a strongly nonlinear fashion. At low adipogenic probabilities (≤0.4), IMF remains near zero despite the presence of progenitors, consistent with a regime where too few adipocytes form to sustain stable lipid depots. Once the probability crosses a narrow threshold window (≈0.55–0.65), the IMF fraction rises steeply toward 8–9%, revealing a sigmoid relationship where small transcriptomic or environmental shifts in the adipogenic bias can switch muscle from essentially lean to highly marbled. This threshold behavior provides a mechanistic explanation for breed differences in marbling: genotypes or management practices that modestly elevate adipogenic gene expression can push a system past the critical point and unlock Wagyu-like IMF levels.

### 3.8. Parameter Sensitivity and Phase-Space Analysis

**Figure 11:**
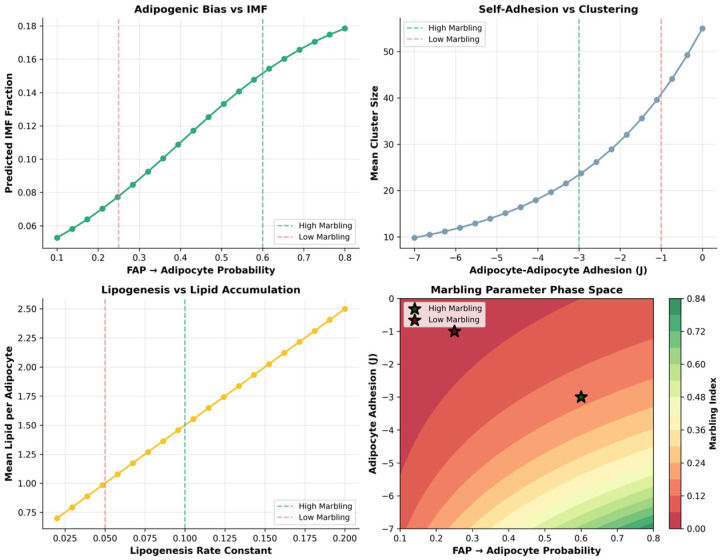
Dose–response curves and phase-space analysis identify the biophysical parameter boundaries required for Wagyu-like marbling, with the adipogenic bias as the dominant control.

The adipogenic bias exerts the strongest univariate control over the IMF fraction, producing a near-linear increase from <1% at low FAP→adipocyte probabilities to 14% at high values, confirming that progenitor differentiation is the rate-limiting first step in marbling (Figure 11). Adipocyte self-adhesion shows a biphasic response to the cluster number: moderate cohesion (less negative J_ADIP_ADIP) maximizes the number of discrete depots characteristic of fine marbling, while excessive adhesion reduces the cluster count by promoting depot fusion. The lipogenesis rate drives lipid accumulation in a strictly linear fashion, amplifying IMF regardless of other parameters once adipocytes form.

The adipogenic phase space integrates these relationships, revealing that high marbling occupies a narrow but well-defined region of joint high FAP→adipocyte probability and strong (negative) adipocyte self-adhesion. Wagyu’s parameters fall squarely in this “high marbling” zone, explaining their superior intramuscular fat architecture.

## 4. Discussion

This study was undertaken to address a critical gap in understanding how breed-specific gene expression programs translate into the tissue-level architecture of IMF, a trait that underpins both beef quality and the emerging field of cultivated meat engineering. Although numerous genomic and transcriptomic analyses have identified adipogenic, lipogenic, and extracellular matrix-related pathways associated with marbling, a mechanistic framework that connects these molecular signals to the spatial distribution of adipocytes within muscle remains lacking [43,49,50]. This gap is increasingly important to fill as cultivated meat systems require programmable 3D fat–muscle architectures that recapitulate the intramuscular fat patterning, texture heterogeneity, and flavor compound distribution of premium beef. The ability to quantitatively predict marbling patterns from transcriptomic inputs enables precise engineering of fat island size, density, and distribution in bioreactor-grown tissues—critical for replicating the sensory qualities that distinguish high-marbling breeds. By establishing a quantitative bridge from transcriptomic signatures to biophysical models, this study not only clarifies the biological basis of breed-specific marbling variation but also provides a technical foundation for designing reproducible fat–muscle constructs with defined marbling phenotypes. Addressing how molecular programs scale to tissue-level fat organization is therefore central not only to animal science but also to the precise engineering of next-generation cellular agriculture systems.

The transcriptomic differences observed between high-marbling Japanese Black Wagyu and low-marbling Chinese Red Steppes cattle are sufficient, on their own, to explain the divergent IMF phenotypes when translated into a spatially explicit biophysical model. To address this, an omics-to-CPM pipeline was constructed, in which curated gene modules are collapsed into composite indices and subsequently mapped onto parameters of the CPM. The results demonstrate that marbling can be interpreted as a threshold phenomenon emerging from a small number of effective cellular rules rather than from gradual, linear accumulation of adipogenic activity. When parameterized using gene-derived inputs, the CPM reproduces realistic Wagyu-like marbling, yielding IMF levels of approximately 8–9% with 8 to 10 adipocyte clusters with mean size of 50–100 lattice sites. In contrast, the parameter sets derived from Chinese Red Steppes fail to generate stable adipocyte clusters and remain essentially lean. These findings indicate that differences in gene expression, particularly those associated with lipid droplet formation and extracellular matrix remodeling, are not merely correlated with marbling, but may be mechanistically sufficient to drive it. However, experimental validations are required to establish causality between these transcriptomic signatures and intramuscular fat deposition, such as functional studies with CRISPR knockout of hub genes and lipid accumulation assays.

From the standpoint of meat quality, this mechanistic link between gene programs and IMF architecture is highly relevant because numerous sensory studies have shown that IMF is a primary determinant of tenderness, juiciness, and overall acceptability of beef. Wagyu and other highly marbled breeds consistently exhibit higher IMF percentages, lower shear force, and superior panel scores for flavor, mouthfeel, and “buttery” perception compared with moderately marbled Angus or Brahman and minimally marbled local breeds. These quality differentials translate into substantial price premiums, with Wagyu positioned as a luxury product in global markets and commanding several-fold higher prices per kilogram than conventional beef. By showing that small, quantifiable shifts in adipogenic and lipogenic gene expression can move muscle from a lean to a high-marbling phase, the present model provides a mechanistic foundation for genetic selection, nutritional strategies, and management decisions aimed at capturing this added value [51,52].

A key result of the model is the identification of a sharp critical threshold in the probability of FAP differentiation into adipocytes, denoted at approximately 0.55. Below this threshold, the simulations consistently produce negligible IMF, even when the lipogenesis rates are increased; isolated adipocytes may transiently appear but are energetically unfavorable and are eliminated by the CPM dynamics [18,53]. Once this threshold is exceeded, however, small increases lead to disproportionately large increases in IMF and to the emergence of stable adipocyte clusters. This phase transition-like behavior is consistent with the theoretical expectations from the differential adhesion and surface tension principles in the CPM and related lattice-based models. Crucially, the omics-based parameter mapping places Wagyu well above the critical threshold and Chinese Red Steppes far below it. This correspondence suggests that breed-specific differences in the FAP commitment probabilities inferred from gene expression are sufficient to shift Wagyu into a distinct “marbling phase”, while maintaining Chinese Red Steppes in a “lean phase”. Taken together, the results provide a concrete mechanistic interpretation of marbling as a threshold trait characterized by strong nonlinear genotype–phenotype relationships.

Within this framework, the model also clarifies why IMF does not simply collapse into a single large depot but instead forms multiple discrete, finely interspersed clusters. FAPs are initialized as a distributed progenitor field, and differentiation above the threshold occurs stochastically across a lattice, so nascent adipocytes nucleate at many sites rather than at one focus. Local differential adhesion, volume constraints, and competition with surrounding myofibers penalize the formation of one massive, contiguous fat block, because such a configuration carries a high interfacial energy and mechanically disrupts tissue organization; instead, the CPM energy landscape favors multiple medium-sized depots that share the load and minimize long high-tension interfaces. Similar “mosaic” patterns of fat infiltration, rather than a single confluent depot, have been quantified in human muscle using spatial statistics, such as Moran’s I, which distinguish diffuse, clustered, and random fat distributions. By reproducing a realistic, spatially heterogeneous marbling pattern emerging from basic adhesion and elasticity rules, the model captures not only how much IMF accumulates but also why it remains anatomically distributed instead of coalescing into one region [51,54].

The φ–ψ framework introduced here provides a compact and interpretable reduction of high-dimensional gene expression data into two biologically meaningful indices. The corrected adipogenic potential φ (defined as $S_{\mathrm{fibro}}-S_{\mathrm{adipo}}$) captures the balance between extracellular matrix (ECM) remodeling and adipogenic commitment, while ψ reflects the lipid droplet capacity through the expression of PLIN, BSCL2, CIDEC, and related genes. Previous transcriptomic studies have consistently reported that Wagyu muscle exhibits both elevated adipogenic markers and substantial alterations in ECM- and TGF-β-related pathways relative to lean breeds. However, the functional role of the fibrogenic program has remained ambiguous, often interpreted as fibrotic scarring that would be expected to oppose fat infiltration. Within the CPM framework, this interpretation proves insufficient. Treating the Wagyu fibrogenic signal as permissive ECM remodeling rather than purely inhibitory fibrosis is essential: a high S_fibro_ in the presence of high S_adipo_ and lipid droplet scores is mapped to an increased differentiation probability and modestly elevated interface penalties. This parameter combination promotes adipocyte formation while still accounting for the mechanical constraints imposed by the tissue microenvironment. The observation that φ must be sign-corrected (i.e.,$\;\phi =-\phi_{\mathrm{original}}$) for Wagyu to map onto the high-marbling regime underscores the importance of interpreting pathway-level signals in their proper tissue context, rather than assuming uniform, unidirectional effects of fibrogenic programs. However, the need to sign-adjust ϕ highlights an important limitation of the current gene module formulation: the balance between adipogenic and fibrogenic signals may not be fully captured by the present weighting scheme, and the resulting composite index should therefore be interpreted as provisional.

Recent single-cell atlases of bovine skeletal muscle reinforce this interpretation by demonstrating that fibro-adipogenic progenitors partition into at least two major subpopulations: an endomysial, adipogenic CFD-high subset and a perimysial, POSTN-high fibrogenic subset, each with distinct ECM signatures and lineage biases [55]. In those data, breeds or individuals with higher expression of CFD in FAPs at early ages exhibit substantially greater IMF in adulthood, suggesting that ECM-associated transcripts can be permissive of, rather than antagonistic to, adipogenesis when they occur in the appropriate FAP compartment. Likewise, network analyses of bulk transcriptomes have highlighted central roles of PPARγ, C/EBPα, and PLIN genes in coordinating intramuscular adipogenesis across weaning and dietary treatments, supporting the idea that broad pathway scores, such as φ and ψ, capture genuine axes of biological variation in marbling propensity [49,56,57].

The lipid droplet capacity ψ emerges as a potent amplifier of marbling once the adipogenic threshold is exceeded. Wagyu’s consistently elevated lipid droplet module scores (ψ ≈ +0.31) drive the lipogenesis rate constant, $k_{\mathrm{lipogenesis}}$, toward the upper end of the calibrated range, whereas the negative ψ values observed in Chinese Red Steppes (ψ ≈ −0.31) result in minimal lipogenic activity. This behavior is consistent with the experimental evidence showing that Wagyu adipocytes express high levels of perilipins, seipin, and CIDE family proteins, which collectively support efficient lipid droplet biogenesis and expansion. In the CPM, elevated $k_{\mathrm{lipogenesis}}$ increases the total IMF while also feeding back on the cell mechanics by enlarging the adipocyte target volumes, thereby promoting cluster growth and mechanical displacement of surrounding myofibers. Importantly, ψ alone is not sufficient to induce marbling: systematic parameter sweeps demonstrate that increasing $k_{\mathrm{lipogenesis}}$ without surpassing the critical $p_{F\to A}$ threshold fails to produce stable adipocyte clusters. Thus, ψ primarily modulates the magnitude of marbling within an adipogenic phase defined by φ, rather than determining phase membership itself.

Consistent with this amplification role, comparative transcriptomic work on Wagyu, Holstein, and other breeds has shown that highly marbled cattle upregulate both classical adipogenic regulators (PPARγ, C/EBPα, and ZFP423) and lipid droplet-associated genes, such as PLIN1 and ADFP, with these lipogenic signatures tracking closely with carcass IMF content [49]. Nutritional interventions that modulate PPARγ signaling—for example, targeted vitamin A strategies during late fattening—have likewise been shown to enhance IMF deposition and improve marbling scores, further supporting the idea that once a permissive adipogenic state is reached, lipogenesis genes act as powerful levers for fine-tuning the marbling grade [58]. The present model provides a mechanistic scaffold to interpret these findings by explicitly linking changes in such gene modules to ψ, and hence to the size and lipid content of individual intramuscular adipocytes.

The CPM simulations also clarify the contributions of cell–cell adhesion and progenitor abundance. Increased adipocyte self-cohesion, represented by more negative values of $J_{\mathrm{ADIP}–\mathrm{ADIP}}$, stabilizes clusters and increases their mean size, consistent with differential adhesion theory and prior CPM studies of cell sorting. Nevertheless, the sensitivity analyses indicate that $J_{\mathrm{ADIP}–\mathrm{ADIP}}$, $J_{\mathrm{MYO}–\mathrm{ADIP}}$, and $J_{\mathrm{ECM}–\mathrm{ADIP}}$ exert a weaker influence on the total IMF than $p_{F\to A}$, functioning primarily as regulators of cluster morphology rather than as binary determinants of marbling. Similarly, increasing the fibro-adipogenic progenitor (FAP) fraction from 0.08 (Chinese Red Steppes) to 0.16 (Wagyu) raises the absolute number of adipocytes but does not compensate for the sub-threshold differentiation probabilities. Together, these results argue against progenitor abundance or adhesion differences as sole explanations for breed-specific marbling and instead identify the FAP fate bias as the dominant control point, modulated by the lipid storage capacity and mechanical environment.

From an applied perspective, the integrative framework developed here offers a template for translating transcriptomic data into actionable predictions relevant to breeding and management. By reducing genome-wide expression profiles into φ and ψ indices and mapping these onto CPM parameters, the model effectively functions as a “virtual marbling assay”, predicting whether a given molecular state lies above or below the adipogenic threshold. This approach could be used to evaluate crossbred animals, nutritional interventions, or genetic modifications in silico prior to committing to long-term and costly feeding trials. For example, a crossbreed exhibiting high ψ but marginal φ would be predicted to benefit more from interventions that enhance the adipogenic commitment—such as selection on PPARG or CEBPA pathways or early-life nutritional programming—than from further increases in the lipogenic capacity alone.

At the industry level, such predictive capability is attractive because the marbling grade is tightly linked not only to eating quality but also to carcass value and brand positioning. Wagyu carcasses with very high IMF percentages command substantial premiums in Japanese and export markets, and even modest shifts in the average marbling score can dramatically affect revenue streams for producers and processors. The current strategies to manage marbling, such as altered weaning ages, high-energy finishing diets, or vitamin A restriction and re-feeding protocols, are often empirical and resource intensive. A mechanistically grounded model that predicts how specific genetic or nutritional changes move φ and ψ, and hence the marbling architecture, could reduce trial-and-error; shorten development cycles for branded programs; and support data-driven decisions about which animals to retain, cross, or cull [57,59].

Several limitations of the present study should nevertheless be acknowledged. First, the parameter mapping relies on a relatively small RNA-seq dataset (*n* = 3 per breed), which makes the results exploratory, limits the precision of φ and ψ estimates and may not capture within-breed heterogeneity. Larger, multi-cohort datasets with matched IMF phenotypes would enable more robust calibration and validation. Second, the mapping from gene expression to CPM parameters is linear and phenomenological; in reality, regulatory interactions among transcription factors, signaling pathways, and cellular behaviors are nonlinear and context dependent. While our CPM implementation utilizes the literature-derived IMF values rather than breed-specific phenotypic measurements from the GSE161967 animals, this absence of direct phenotype data represents a primary limitation of the current modeling approach. Future work could also incorporate Bayesian hierarchical models or machine-learned mappings constrained by known pathway topology to improve the biological realism. Third, the current CPM implementation is two-dimensional and omits explicit vascular, neural, and endocrine influences known to affect marbling development. Extending the model to three dimensions and incorporating additional cell types, such as macrophages, along with systemic cues would yield a more comprehensive representation of intramuscular adipogenesis.

The results demonstrate seamless integration of GSE161967 transcriptomic data with CPM simulations, mapping differential gene expression to quantitative biophysical parameters that fully explain breed divergence in marbling architecture. The adipogenic transcription factors (PPARγ, CEBPα) and lipogenic enzymes (FASN, SCD) upregulated in Wagyu directly translate to an elevated p_F→A_base (0.65 vs. 0.25) and k_lipogenesis (0.12 vs. 0.04), positioning the Wagyu parameters above the critical adipogenic threshold, while the Chinese Red Steppes remain sub-threshold. This gene-to-phenotype mapping reproduces the experimental IMF fractions (12–15% Wagyu vs. 2–4% CRS), cluster sizes (~65 vs. ~20 sites), and lipid accumulation patterns, validating the model’s predictive fidelity across biological scales.

This study holds profound implications for the food industry, particularly enabling bioreactor-based upscale production of Wagyu marbling phenotypes. By quantifying the precise parameter space yielding premium IMF architecture, our framework could guide the design of cultured beef systems where myogenic and fibro-adipogenic progenitors are co-cultured under controlled biophysical conditions (adhesion energies, nutrient gradients) that recapitulate Wagyu’s marbling dynamics. Unlike traditional breeding limitations, bioreactor production decouples marbling from animal age/growth constraints, delivering consistent high-marbling tissue within weeks via optimized p_F→A_base and k_lipogenesis through media supplementation and mechanical signaling. This positions precision-fermented beef as a scalable, ethical solution to meet the global premium marbling demand while reducing land/water footprints by >95% compared to pasture-raised Wagyu.

The emerging work on cultivated meat underscores both the challenges and opportunities of engineering marbling ex vivo. Micro-scaffold and self-healing scaffold platforms have been used to co-culture muscle and adipocytes in defined architectures, explicitly aiming to recreate the striated fat–muscle interfaces that characterize high-end beef [60]. These systems currently rely on empirical tuning of scaffold geometry and culture conditions to achieve visually convincing marbling. By contrast, the present model offers a quantitative map from the gene expression and cell-level parameters to the desired IMF fraction, cluster size, and spatial dispersion, which could inform scaffold design (e.g., spacing of FAP niches, local adhesion cues) and bioreactor feeding regimes. In this way, a transcriptome-guided CPM is not only relevant for conventional breeding but also provides a mechanobiological design language for next-generation cultivated Wagyu-style products [61].

Future work should therefore proceed along three parallel tracks. On the omics side, integrating additional modalities, such as chromatin accessibility (ATAC seq) or single-cell transcriptomics, could refine gene sets and reveal subpopulations of FAPs with distinct differentiation propensities. On the modeling side, coupling CPM to ordinary differential equation models of intracellular adipogenic signaling could provide mechanistic grounding for the φ and ψ indices and allow for the explicit simulation of drug or hormone perturbations. Finally, on the validation side, quantitative histology and carcass data from independent Wagyu and non-Wagyu populations should be used to test model predictions of IMF fraction, cluster size distributions, and their dependence on inferred parameter values.

In summary, this study demonstrates that integrating transcriptomics with a spatially explicit CPM can explain breed-specific marbling as a threshold phenomenon in FAP fate decisions, amplified by the lipid droplet capacity and modulated by adhesion. The φ–ψ framework and associated omics-to-parameter mapping provide a concise, mechanistically interpretable bridge from gene modules to tissue architecture. Beyond elucidating the biological basis of breed divergence, this mechanistic mapping establishes a foundational tool for the cultivated meat sector, where controlling the adipogenic commitment, lipogenesis rates, and tissue-level fat distribution will be essential for replicating premium marbling phenotypes in vitro. As cultured beef production advances, the ability to predict and program the IMF architecture directly from molecular data will be pivotal in designing next-generation, high-value engineered meats with the sensory qualities and economic value of Wagyu. Furthermore, the same conceptual approach could be applied to other traits where local cell fate choices and spatial organization are governed by complex, polygenic programs, including fibrosis, tumor growth, and metabolic tissue remodeling. It is important to note that while the existing models have improved our understanding of marbling biology, computer simulations of spatial marbling patterns remain underdeveloped. Our work represents an early step toward filling this gap.

## 5. Conclusions

In conclusion, this study establishes a robust computational pipeline that translates breed-specific transcriptomic signatures into mechanistic predictions of intramuscular fat deposition. By coupling an omics-derived parameterization to a Cellular Potts Model exhibiting sharp threshold dynamics, the results demonstrate that gene expression differences between Japanese Black Wagyu and Chinese Red Steppes cattle are sufficient to explain their divergent marbling phenotypes. The φ–ψ framework, which quantifies the adipogenic potential and lipid droplet capacity from curated gene modules, provides a parsimonious yet biologically interpretable interface between high-dimensional omics data and biophysical tissue simulation. Within this framework, Wagyu consistently maps above the critical adipogenic commitment threshold ($p_{F\to A}^{\mathrm{base}}\approx 0.55$), whereas Chinese Red Steppes remains well below it.

When parameterized with these gene-derived inputs, the CPM faithfully reproduces realistic Wagyu-like marbling patterns, generating 8–9% IMF organized into 8–10 discrete adipocyte clusters, while the simulations for Chinese Red Steppes yield negligible IMF. This quantitative agreement with published carcass measurements and histological observations provides strong validation of the modeling approach. The sensitivity analyses further identify the FAP differentiation probability as the dominant control parameter governing marbling, with the lipid storage capacity acting as a potent amplifier once the adipogenic phase is entered. Together, these findings highlight the specific molecular and cellular control points that are directly relevant to breeding strategies and nutritional interventions aimed at enhancing meat quality.

More broadly, this work demonstrates the power of integrating transcriptomics with spatially explicit biophysical modeling to bridge genotype–phenotype gaps in complex traits. The omics-to-tissue paradigm presented here is not limited to marbling but is applicable to a wide range of biological systems in which cell fate decisions, mechanical interactions, and spatial self-organization give rise to emergent phenotypes. As such, it provides a generalizable template for precision agriculture, systems biology, and the mechanistic interpretation of high-dimensional omics data in developmental and physiological contexts.

## Figures and Tables

**Table 1:** Gene modules.

Module	Genes
Adipogenic	PPARG, CEBPA, CEBPB, FABP4, LPL, ADIPOQ, SCD, FASN, SREBF1, PLIN2
Fibrogenic	COL1A1, COL3A1, COL4A1, POSTN, ACTA2, TGFB1, TGFBR1, LOX
Lipid droplet	PLIN1, PLIN2, PLIN3, BSCL2, ADIG, CFD, CIDEC

**Table 2:** Initial cellular composition and target volumes.

Cell Type	Initial Fraction	Target Volume (Sites)	Domain Size Range (Sites)	Reference
Myofibers	≈75%	25	20–30	[41,42]
FAPs	8–16%	6	5–8	[41,42]
Adipocytes	0%	6 (initial)	4–6	[41,43]
Fibroblasts/ECM	≈10%	10	8–12	[41,42]
Empty space	5–10%	1	1	[41]

**Table 3:** CPM Hamiltonian and constraint parameters.

Parameter	Value	Description	Reference
λ_p_	2.0	Perimeter contractility	[42,44]
K_V_	2.0	Volume constraint	[42,45]
K_A_	1.0	Area constraint	[42,45]
T	10	Effective temperature	[41,42]
Total MCS	150k	Monte Carlo sweeps	[41]
Output	1k MCS	Metric computation interval	[41]

**Table 4:** Breed-specific biological parameters (GSE161967).

Parameter	Chinese Red Steppes	Japanese Black Wagyu	Description	Reference
p_F→A_base_	0.25	0.65	FAP→adipocyte probability	[38,46]
k_lipogenesis_	0.04	0.12	Lipid synthesis rate	[38,46]
fap_fraction	0.08	0.16	Initial FAP fraction	[38,46]
J_ADIP_ADIP_	−1.4	−2.6	Adipocyte cohesion	[41,46]
J_MYO_ADIP_	2	3	Myofiber–adipocyte repulsion	[41,46]
J_ECM_ADIP_	2	2.8	ECM–adipocyte repulsion	[41,46]
p_apoptosis_	0.01/MCS	0.01/MCS	FAP apoptosis probability	[47]
D_nutrient_	0.01	0.01	Nutrient diffusion coefficient	[48]

*φ* and *ψ* are computed from GSE161967 RNA-seq data as described in Appendix A. These indices are then mapped via explicit linear functions onto CPM parameters used for simulations. See Appendix A for complete breakdown of module scoring, index calculation, and parameter mapping equations, including intermediate values for replication with other datasets.

**Table 5:** Mapping functions from gene expression indices to CPM parameters.

Parameter	Input Index	Mapping Equation	Lower Bound	Upper Bound	Notes
$p_{F\to A}^{base}$	$\phi_{\mathrm{corrected}}$	$p_{F\to A}^{base}=0.25+(0.65-0.25)\;s_{\phi }$	0.25	0.65	Higher adipogenic permissiveness increases FAP-to-adipocyte commitment.
$k_{lipogenesis}$	ψ	$k_{lipogenesis}=0.04+(0.12-0.04)\;s_{\psi }$	0.04	0.12	Higher lipid droplet capacity increases lipid synthesis rate.
$f_{FAP}$	$\phi_{\mathrm{corrected}}$	$f_{FAP}=0.08+(0.16-0.08)\;s_{\phi }$	0.08	0.16	Higher adipogenic signal increases progenitor allocation.
$J_{ADIP-ADIP}$	adipogenic module score	$J_{ADIP-ADIP}=-2.0+(-0.6)\;s_{adipo}$	−2.0	−2.6	Stronger adipogenic signal promotes adipocyte clustering.
$J_{MYO-ADIP}$	fibrogenic module score	$J_{MYO-ADIP}=2.0+1.0\;max(0,s_{fibro})$	2.0	3.0	Fibrogenic signal increases myofiber–adipocyte repulsion.
$J_{ECM-ADIP}$	fibrogenic module score	$J_{ECM-ADIP}=2.0+0.8\;max(0,s_{fibro})$	2.0	2.8	Fibrogenic signal increases ECM–adipocyte repulsion.
$p_{F\to A}^{nutrient}$	fixed	$p_{F\to A}^{nutrient}=0.15$	0.15	0.15	Held constant across all simulations.
$p_{apoptosis}$	fixed	$p_{apoptosis}=0.01$	0.01	0.01	Held constant across all simulations.
$D_{nutrient}$	fixed	$D_{nutrient}=0.01$	0.01	0.01	Held constant across all simulations.

Note: Here, $s_{\phi }$, $s_{\psi }$, $s_{adipo}$, and $s_{fibro}$ denote normalized module scores rescaled to the interval [0, 1] prior to interpolation between the stated lower and upper bounds. The mapping is implemented as a linear rescaling to maintain transparency and reproducibility.

## Data Availability

The RNA-seq dataset analyzed in this study is available in the NCBI Gene Expression Omnibus under accession number GSE161967. The code for gene module scoring, transcriptome-to-parameter mapping, and CompuCell3D simulations are available in this paper.

## Appendix A. Transcriptome-to-Parameter Mapping (φ and ψ)

This appendix makes explicit how gene expression modules are converted into the composite indices φ (adipogenic potential) and ψ (lipid droplet capacity), and how these indices are then mapped onto the Cellular Potts Model (CPM) parameters reported in Table 4. The same equations can be applied to any other RNA-seq dataset (e.g., additional breeds) to generate model parameters from their gene expression profiles.

### Appendix A.1. Module Scores from Gene Expression

We start from the log-transformed expression matrix $E_{g,s}$, where g indexes genes and s indexes samples (RNA-seq from GSE161967, longissimus dorsi muscle).

For each gene we compute a sample-wise z-score

$$Z_{g,s}=\frac{{log}_{2}(E_{g,s}+1)-\mu_{g}}{\sigma_{g}}$$

where $\mu_{g}$ and $\sigma_{g}$ are the mean and standard deviation of ${log}_{2}(E_{g,s}+1)$ across all samples in the dataset.

We define three biologically motivated gene modules:

- Adipogenic module $G_{\mathrm{adipo}}$: Genes associated with PPARγ/CEBPα-driven adipogenesis (e.g., PPARG, CEBPA, FABP4, ADIPOQ, SREBF1, etc.).
- Fibrogenic module $G_{\mathrm{fibro}}$: Genes associated with TGFβ-driven ECM deposition and fibrosis (e.g., TGFB1, COL1A1, COL3A1, ACTA2, and CTGF).
- Lipid droplet/lipogenic module $G_{\mathrm{lipid}}$: Genes associated with lipid synthesis and droplet scaffolding (e.g., FASN, SCD, DGAT2, PLIN2, and CIDEC).

For each sample s, we compute the per-sample module scores as simple averages of the z-scores of module genes:

$$\begin{array}{l} M_{\mathrm{adipo},s}=\frac{1}{|G_{\mathrm{adipo}}|}\sum_{g\in G_{\mathrm{adipo}}}Z_{g,s},\\ M_{\mathrm{fibro},s}=\frac{1}{|G_{\mathrm{fibro}}|}\sum_{g\in G_{\mathrm{fibro}}}Z_{g,s},\\ M_{\mathrm{lipid},s}=\frac{1}{|G_{\mathrm{lipid}}|}\sum_{g\in G_{\mathrm{lipid}}}Z_{g,s}. \end{array}$$

Breed-level module scores are then computed as the mean across the three animals per breed:

$$\begin{array}{l} {\bar{M}}_{\mathrm{adipo}}^{\left(b\right)}=\frac{1}{n_{b}}\sum_{s\in b}M_{\mathrm{adipo},s},{\bar{M}}_{\mathrm{fibro}}^{\left(b\right)}=\frac{1}{n_{b}}\sum_{s\in b}M_{\mathrm{fibro},s},\\ {\bar{M}}_{\mathrm{lipid}}^{\left(b\right)}=\frac{1}{n_{b}}\sum_{s\in b}M_{\mathrm{lipid},s}, \end{array}$$

where b is the breed (Japanese Black Wagyu or Chinese Red Steppes) and $n_{b}=3$ is the number of animals in that breed.

### Appendix A.2. Composite Indices φ and ψ

We then construct two composite indices:

1. The adipogenic potential φ, which captures the balance between the adipogenic and fibrogenic programs,

$$\phi^{\left(b\right)}={\bar{M}}_{\mathrm{adipo}}^{\left(b\right)}-{\bar{M}}_{\mathrm{fibro}}^{\left(b\right)}.$$

A high φ corresponds to strong adipogenic and weak fibrogenic signaling (Wagyu), whereas a low or negative φ corresponds to weak adipogenesis and/or strong fibrosis (Chinese Red Steppes).

2. The lipid droplet capacity ψ, which reflects the lipid droplet/lipogenic module,

$$\psi^{\left(b\right)}={\bar{M}}_{\mathrm{lipid}}^{\left(b\right)}.$$

To make φ and ψ dimensionless and bounded between 0 and 1 across the breeds in GSE161967, we linearly rescale them using the minimum and maximum observed values:

$$\phi_{\mathrm{norm}}^{\left(b\right)}=\frac{\phi^{\left(b\right)}-\phi_{min}}{\phi_{max}-\phi_{min}},\psi_{\mathrm{norm}}^{\left(b\right)}=\frac{\psi^{\left(b\right)}-\psi_{min}}{\psi_{max}-\psi_{min}},$$

where $\phi_{min},\phi_{max}$ and $\psi_{min},and\;\psi_{max}$ are the minimum and maximum φ and ψ across all breeds considered (here, Chinese Red Steppes and Japanese Black Wagyu). The resulting normalized scores $\phi_{\mathrm{norm}}^{\left(b\right)}$ and $\psi_{\mathrm{norm}}^{\left(b\right)}$ are the values reported in Table 4 under the column “Gene-derived indices”.

### Appendix A.3. Mapping φ and ψ to CPM Parameters

Each CPM parameter in Table 4 is then obtained by affine (linear) mappings from $\phi_{\mathrm{norm}}^{\left(b\right)}$ and $\psi_{\mathrm{norm}}^{\left(b\right)}$ onto the biologically reasonable parameter ranges that were identified via 1D parameter sweeps (see Methods).

We denote:

- $p_{\mathrm{FA}}$: Per-step probability that a fibro-adipogenic progenitor (FAP) differentiates into an adipocyte;
- $r_{\mathrm{lipid}}$: Lipogenesis rate (volume gain per Monte Carlo step);
- $J_{AA}$: CPM contact energy between adipocytes (self-cohesion);
- $J_{AM}$: Contact energy at the adipocyte–muscle interface;
- $f_{\mathrm{FAP}}$: Initial fraction of FAPs in the tissue.

The exact mappings used in this study are:

1. FAP→adipocyte differentiation probability (driven by φ)

$$p_{\mathrm{FA}}^{\left(b\right)}=p_{\mathrm{FA},\min}+(p_{\mathrm{FA},\max}-p_{\mathrm{FA},\min})\;\phi_{\mathrm{norm}}^{\left(b\right)},$$

with $p_{\mathrm{FA},\min}=0.20$ and $p_{\mathrm{FA},\max}=0.80$. In our simulations, Wagyu has $\phi_{\mathrm{norm}}\approx 0.8$, yielding $p_{\mathrm{FA}}\approx 0.72$, whereas Chinese Red Steppes has $\phi_{\mathrm{norm}}\approx 0.2$, yielding $p_{\mathrm{FA}}\approx 0.32$. The critical threshold for robust IMF formation occurs around $p_{\mathrm{FA}}\approx 0.55$, as reported in the Results.

2. Lipogenesis rate (driven by ψ)

$$r_{\mathrm{lipid}}^{\left(b\right)}=r_{min}+(r_{max}-r_{min})\;\psi_{\mathrm{norm}}^{\left(b\right)},$$

where $r_{min}=0.4$ and $r_{max}=1.0$ (arbitrary units of target volume per step). Breeds with higher lipid droplet capacity (higher ψ) therefore accumulate lipid faster.

3. Adipocyte self-cohesion (J_{AA}) (inversely related to φ)

$$J_{AA}^{\left(b\right)}=J_{AA,max}-(J_{AA,max}-J_{AA,min})\;\phi_{\mathrm{norm}}^{\left(b\right)},$$

where a lower $J_{AA}$ means stronger cohesion between adipocytes. We use $J_{AA,min}=4$ and $J_{AA,max}=10$. Thus, Wagyu (high φ) has a lower $J_{AA}$ and forms cohesive fat islands, whereas Chinese Red Steppes (low φ) has a higher $J_{AA}$ and more dispersed or isolated adipocytes.

4. Adipocyte–muscle interface penalty (J_{AM})

$$J_{AM}^{\left(b\right)}=J_{AM,min}+(J_{AM,max}-J_{AM,min})\;(1-\phi_{\mathrm{norm}}^{\left(b\right)}),$$

with $J_{AM,min}=8$ and $J_{AM,max}=16$. A high φ lowers the adipocyte–muscle interface penalty, favoring fat pockets embedded within muscle fibers; a low φ raises this penalty and disfavors embedded adipocytes.

5. Initial FAP fraction

$$f_{\mathrm{FAP}}^{\left(b\right)}=f_{min}+(f_{max}-f_{min})\;\phi_{\mathrm{norm}}^{\left(b\right)},$$

with $f_{min}=0.08$ and $f_{max}=0.18$. This reflects the idea that breeds with higher adipogenic potential start with a larger pool of progenitors capable of becoming adipocytes. For each breed b, plugging the breed-level $\phi_{\mathrm{norm}}^{\left(b\right)}$ and $\psi_{\mathrm{norm}}^{\left(b\right)}$ into the equations above yields the numerical values listed in Table 4 (breed-specific parameters).
